# Partial EC outputs by degraded cues are amplified in hippocampal CA3 circuits for retrieving stored patterns

**DOI:** 10.1371/journal.pone.0281458

**Published:** 2023-04-19

**Authors:** Kisang Eom

**Affiliations:** Department of Physiology, School of Medicine, Keimyung University, Dalseogu District, Daegu Metropolitan City, Republic of Korea; University of Modena and Reggio Emilia, ITALY

## Abstract

Hippocampus is known to be important for episodic memories. Measuring of hippocampal neural ensembles is therefore important for observing hippocampal cognitive processes such as pattern completion. Previous studies on pattern completion had a limitation because the activities of CA3 were not simultaneously observed with the activities of the entorhinal cortex that project to the CA3. In addition, in previous research and modelling, distinct concepts such as pattern completion and pattern convergence have not been considered separately. Here, I used a molecular analysis technique that enables comparison of neural ensembles that evoked two successive events and evaluated neural ensembles in the hippocampal CA3 region and entorhinal cortex. By comparing neural ensembles in hippocampus and entorhinal cortex, I could obtain evidence that suggests pattern completion occurring in the CA3 region was induced by the partial input from EC. Use of the molecular-based ensemble measurement allows measuring two or more brain regions simultaneously, which can lead to insights into the cognitive functions of neural circuits.

## Introduction

The hippocampus has an important role in the encoding and retrieval of episodic memory [1]. For the encoding and retrieval of episodic memory, two distinct process would be important: pattern separation and pattern completion. Pattern separation is a process in neural networks that causes redundant or similar inputs to be converted into less similar outputs, which is known that the dentate gyrus (DG) of hippocampus that constitutes the sparse network contributes this phenomenon. Pattern completion is another process in neural network that reconstructs of complete stored representations from partial inputs that are part of stored representation [2]. Since previous literatures had suggested that auto-associational networks of hippocampal CA3 regions would contribute to pattern completion and associative memory [3–6], many studies for pattern completion have been conducted [7–10]. Previous studies revealed that the layer II of entorhinal cortex (EC) sends axon to distal dendrites of CA3 pyramidal cells (CA3-PCs) and dendrites of granule cells of dentate gyrus (DG) [4]. Recently, a study revealed that the hippocampus converts dynamic activities from the EC into stable ones [11]. Therefore, in order to study the activities of the hippocampus related to behavioral tasks, it would be necessary to simultaneously consider the activities of the hippocampus and EC.

Previously, EC ensembles or hippocampal CA3 ensembles have been measured using various methods, including *in vivo* electrophysiology or live imaging [11–13]. However, studies to observe the activity of the hippocampus and EC at the same time have only recently begun [11]. In previous studies, pattern completion is regard as a situation in which the similarity between output ensembles is greater than that between input ensembles [9, 13, 14]. However, this situation corresponds to pattern convergence, not pattern completion [15]. Considering the previous models of pattern completion [6, 15] and the influence of EC neural activity on CA3 neural activity [11, 16, 17], it would be important to compare the EC activities evoked by either the partial external input or whole external input with the activities of CA3 corresponding to each of the two activities. However, in previous experiments on pattern completion, the activity of the EC responsible for projecting axons to CA3 via the PP [18, 19] has been often overlooked.

Here, I applied H1a/Arc catFISH (cellular analysis of temporal activity of fluorescence of in situ hybridization with *Arc* and *H1a* riboprobes) [20, 21] to measure neural ensembles in EC and CA3 and found that activation of a subset of CA3 ensembles which would be evoked by partial activation of EC ensembles [6, 18, 22] effectively activates the remaining CA3 ensembles. The IEG-based imaging method is a convenience method that can easily measure and compare neural ensembles in multiple regions [20, 23]. With this method, I was able to confirm that pattern completion of CA3 circuits could be triggered by direct cortical input and the storage capacity of CA3 could contribute to the storage and retrieval of stored patterns in neural circuits during rapid contextual learning.

## Materials and methods

### Animals and maintenance procedure

For using animals in experiments, 3-month-old male C57/BL6J mice were maintained in standard environmental conditions (temperature: 25 ± 2°C, humidity: 60 ± 5%, dark/light cycle: 8:00 p.m.–8:00 a.m. of next day/8:00 a.m.–8:00 p.m.) and monitored under the veterinary supervision. All mice used in the experiment were housed alone for 1 week before the experiment, during which time they were acclimatized to routine handling. All animal procedures were approved by the Animal Care Committee of Keimyung University (KM-2021-07).

### Pre-exposure mediated contextual fear conditioning

**Fig 1:**
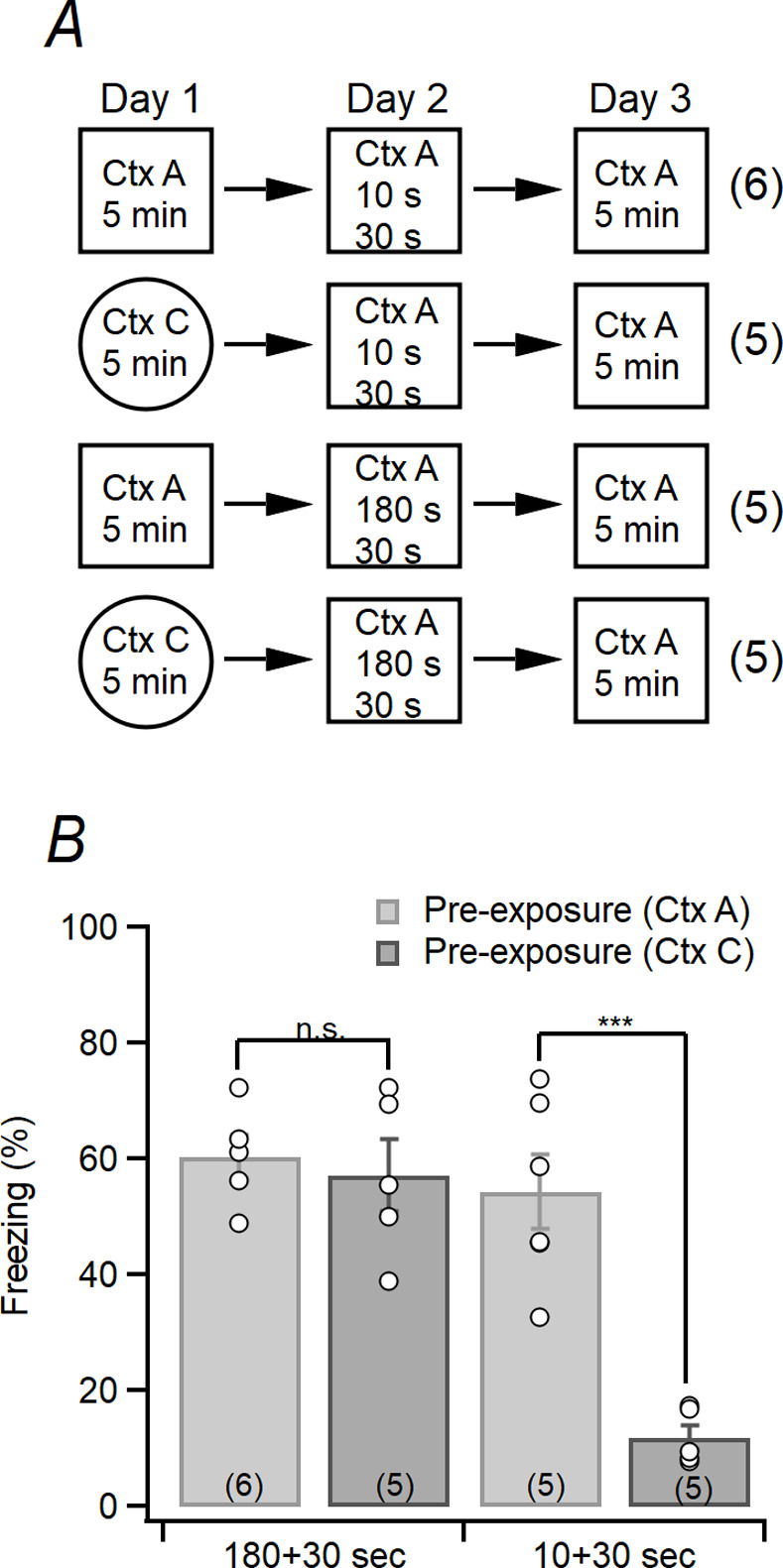
Protocols for pre-exposure mediated contextual fear conditioning (PECFC) and results. (A) Schedule of the PECFC. On the day 1, mice were exposed to either the Ctx A (preexposure) or Ctx C (non-preexposure) for 5 m. On the day 2, the Ctx A group and the Ctx C group were divided into four experimental subgroups according to the place-to-shock interval (10 s and 180 s) as shown in this figure. All mice returned to homecage after 30 s from the shock. On the day 3, freezing of mice was measured during the exposure of Ctx A for 5 m. (B) Based on schedule in the Fig 1A, freezing level of mice exposed to the Ctx A on day 3 was measured (context: F_(1,17)_ = 18.967, p < 0.001; time: F_(1,17)_ = 24.136, p < 0.001; context × time: F_(1,17)_ = 14.122, p = 0.002, GLM).

The Pre-exposure mediated contextual fear conditioning (PECFC) tasks of animals are based on the context pre-exposure facilitation effect (CPFE), i.e., enhanced contextual fear conditioning due to pre-exposure to the context before a separate brief context shock episode [24]. Previous studies have shown that this phenomenon is dependent on hippocampus-dependent conjunctive representation [25] and impaired by ablation of CA3 output [7]. In the evaluation of pattern completion, it will be important to evaluate the degree of degradation to retrieval cues and the degree of the corresponding response of the neural networks [2]. Because pattern completion has been predicted to depend on auto-associational networks of hippocampal region [3, 6], the PECFC protocol could be used for evaluation of pattern completion. For this, 21 mice (15–25 weeks old, either sex) were transported to the experiment room and left undisturbed for 30 min prior to the experiment. Then, mice were exposed either context A (Ctx A) or context C (Ctx C). Ctx A is a chamber (18 cm wide × 18 cm long × 30 cm high; H10-11M-TC; Coulbourn Instruments, PA) consisting of a metal grid floor, aluminum side walls, and a clear Plexiglass front door and back wall, which is lit indirectly with a 12 W light bulb. Context C (Ctx C) consists of a white acrylic blind end cylinder (15 cm in diameter, 18 cm in height, and 0.5 cm in thickness) vertically on the metal grid floor of the conditioning chamber, and covered the bottom inside the cylinder, on which mice were placed. The chamber was cleaned with 70% ethanol between runs. On Day 1, mice were divided into two groups. Each group was allowed to freely explore either Ctx A or C for 5 min (pre-exposure). On Day 2, two groups were divided again into 10-sec-shock and 3-min-shock subgroups. Detailed experimental schedule is summarized in Fig 1A. The 10-sec-shock subgroups were placed into Ctx A for 10 s, received a single foot shock (1 mA, for 2 s) and were returned to their home cages 30 s after the shock. The 3-min-shock subgroups were placed into context A for 180 s, received a single foot shock (1 mA, for 2 s) and were returned to their home cages 30 s after the shock. On Day 3, freezing was assessed by placing the animals in context A for 5 min. The activity of animals was recorded at 30 frames per second and stored as video files. These video files were fed into an open-source video analysis pipeline, ezTrack [26], to assess the freezing of animals. Freezing ratio was calculated as a total duration of freezing (in s) divided by the total duration of observation (300 s). Detailed procedures were based on the previous studies [21].

### Arc/H1a catFISH for ensemble similarity

**Fig 2:**
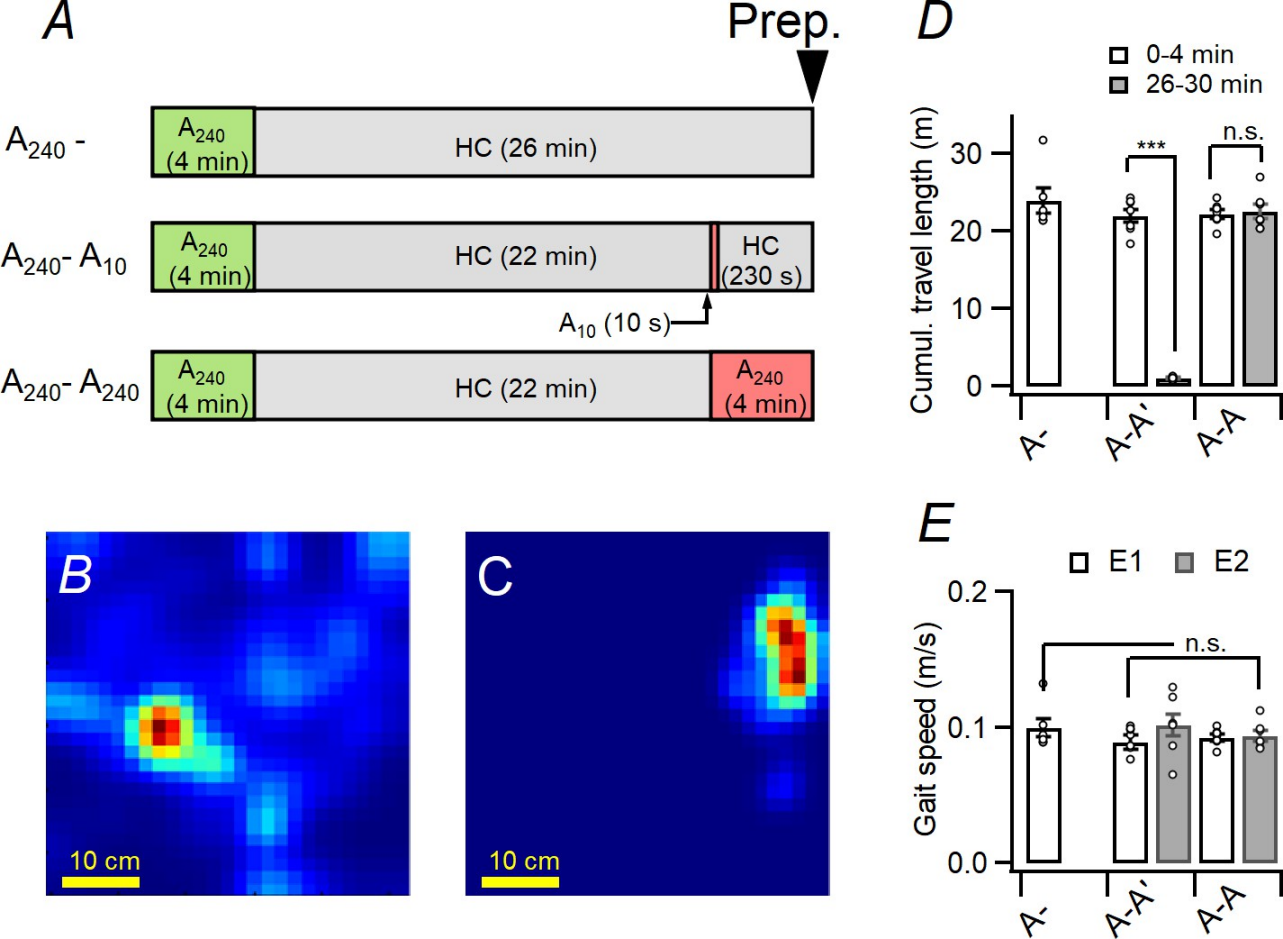
Schematic diagram of contexts and experiment protocol as well as an assessment of animal responses. (A) Schedule of the procedure for cohort A_240_- (*upper*), A_240_-A_10_ (*middle*), and A_240_-A_240_ (*lower*). For the cohort A_240_-, mice experienced A_240_ and returned to HC for 26 min, and sacrificed. For the cohort A_240_-A_10_, mice experienced A_240_ and returned to HC, where they stayed for 22 minutes. After experiencing A_10_, they returned to HC again for 230 seconds and were then sacrificed to obtain brain slices. For the cohort A_240_-A_240_, mice were exposed to the A_240_ twice with 22-min interval and sacrificed to obtain brain slices. (B-C) The heatmap of animal trajectory for A_240_ (Fig 2B) and A_10_ (Fig 2C). (D-E) Cumulative travel length (D) and gait speed (E) of animals in in each context for three cohorts. Note that there is no significant difference of gait speed among the animals.

To investigate how EC and CA3 manages conjunctive representation, I purchased Mm-Homer1a-tvS (Cat# 433941-C2, which detects intranuclear *H1a* mRNA) and Mm-Arc-C3 (Cat# 316911-C3, which detects intranuclear *Arc* mRNA) probes from ACDBio Inc (Newark, CA, USA) and used to observe LEC/MEC and CA3 neuronal ensembles activated by two sequential behavioral experiences with *H1a*/*Arc* catFISH [20]. Previous study revealed that *Arc* mRNA is generated from short transcripts (ca. 3.5 kb), whereas H1a mRNA is generated from long transcripts (ca. 45 kb) [27]. The *Arc* riboprobe and *H1a* riboprobe targets the whole length of Arc mRNA and 3’-untranslated region of H1a mRNA, respectively [23]. Hence, *Arc* riboprobe marks for neurons activated ca. 0–5 min before sacrifice, whereas *H1a* riboprobe marks neurons activated ca. 25–30 min before sacrifice (S1 Fig). Animals were allowed to explore the novel environment, which was cleaned with 70% ethanol between each animal. The novel environment consisted of white acrylic box (60 cm × 60 cm × 60 cm) in a quiet room for 4 min. After resting for 22 min in the homecage (HC), animals were ether continued to stay in the HC or exposed to the novel environments (Fig 2A). A 4-minute stay of the animal in the novel environment described above will be referred to ‘A_240_’, and a 10 s stay in the novel environment described above will be referred as ‘A_10_’. Therefore, based on the context of exposure in the last 26–30 minutes, the animals were divided into three cohorts. The detailed schedules of behavioral procedures for *Arc/H1a* cellular analysis of temporal FISH (hereafter *Arc*/*H1a* catFISH) were as follows (Fig 2A): stay in homecage for the remaining 4 minutes (cohort A_240_-; 6 mice; *upper* of Fig 2A), exposure of the A_10_ (cohort A_240_-A_10_; 7 mice; *middle* of Fig 2A) and exposure of the A_240_ again (cohort A_240_-A_240_; 7 mice; *lower* of Fig 2A). The activity of animals was recorded at 30 frames/s and stored as a video file and evaluated using open-source video analysis pipeline, ezTrack [26]. The animal’s gait speed was calculated as (cumulative distance traveled for elapsed time)/(length of elapsed time). Exploration of animals in novel environment is described as heatmap (A_240_: Fig 2B; A_10_: Fig 2C) and quantified through the mice’s cumulative trajectory distance (Fig 4D) and walking speed (Fig 4E). Immediately after the end of behavioral procedures, mice were decapitated and the brain was removed and cut along the longitudinal fissure to separate two cerebral hemispheres. The harvested brains were quickly frozen in liquid nitrogen and stored at −70°C for further process. Author obtained sagittal tissue sections containing medial/lateral EC (MEC/LEC; mediolateral, ca. ±3.3 mm from longitudinal fissure) or dorsal CA3 (mediolateral, ca. ±2.3 mm from longitudinal fissure). The sections were fixed in 4% PFA for 10 min, dehydrated in increasing concentrations of ethanol (50%, 70%, and 100%) for 5 min, and finally air-dried. The fixed tissues were pretreated for protease digestion for 30 min at room temperature. The digested tissues were hybridized with appropriate *H1a* and *Arc* riboprobes according to instructions in manufacturer’s manual and previous studies and visualized to measure neural ensembles [21, 28]. Nuclei were counterstained with 4′,6-diamidino-2-phenylindole (DAPI; Tocris bioscience, Bristol, UK).

### Kinetics of H1a and Arc riboprobes

**Fig 3:**
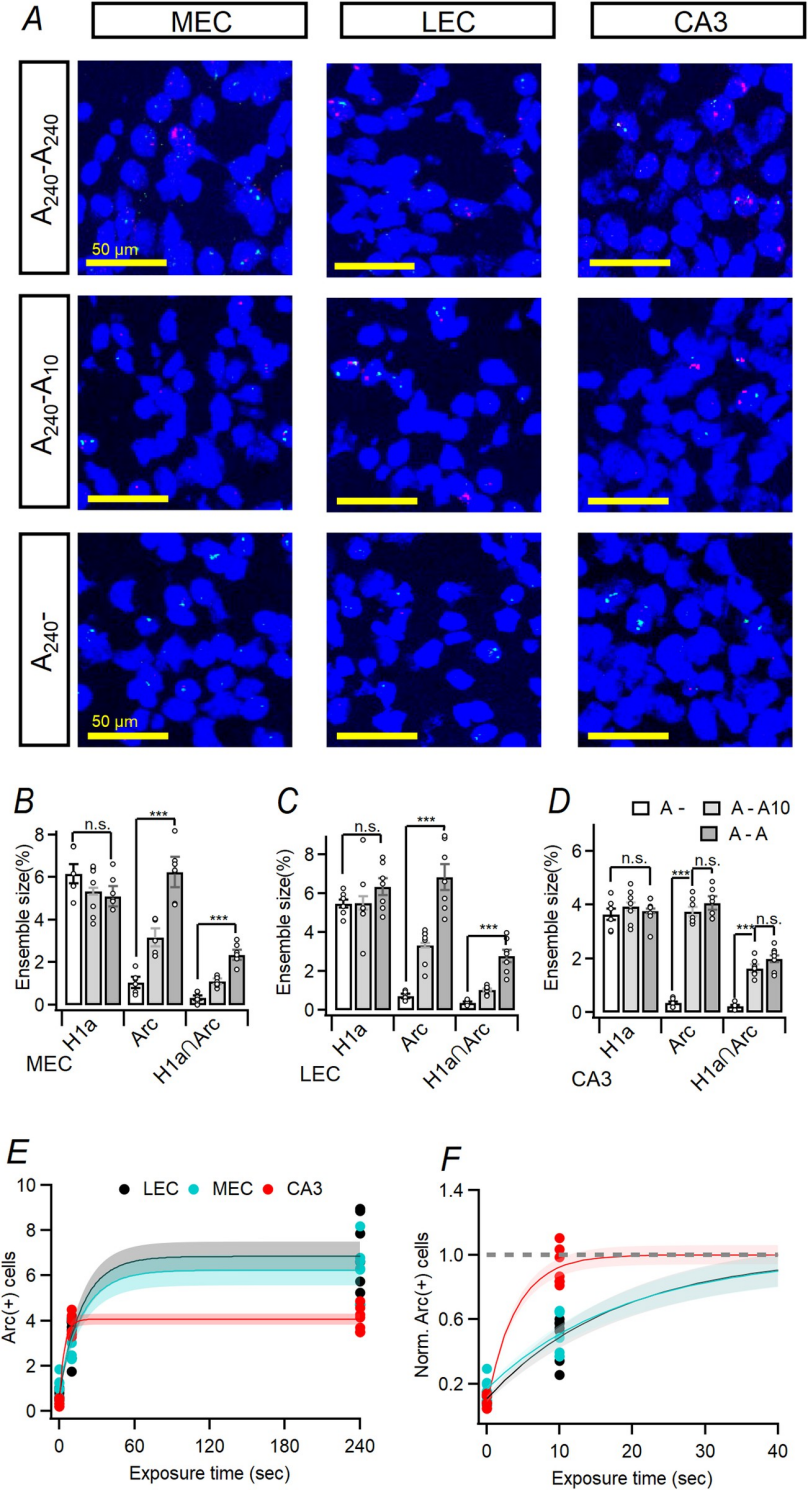
Analysis of ensembles for hippocampal CA3 and LEC/MEC regions. (A) Representative confocal images of H1a (green) and Arc (red) transcripts in nuclei for Layer II of MEC/LEC (*left* column and *middle* column, respectively) and CA3 (right column) from animals of cohort A_240_- (upper row), A_240_-A_10_ (middle row) and A_240_-A_240_ (right row). (B-D) Summary of the *H1a*(+) and *Arc*(+) ensemble size on layer II of MEC (probe: F_(1,34)_ = 47.847, p < 0.001, cohort: F_(2,34)_ = 17.047, p < 0.001, probe × cohort: F_(2,34)_ = 32.721, p < 0.001; GLM; Fig 3B), LEC (probe: F_(1,34)_ = 29.287, p < 0.001, cohort: F_(2,34)_ = 26.273, p < 0.001, probe × cohort: F_(2,34)_ = 14.481, p < 0.001; GLM; Fig 3C) and CA3 (probe: F_(1,34)_ = 43.595, p < 0.001, cohort: F_(2,34)_ = 58.828, p < 0.001, probe × cohort: F_(2,34)_ = 46.858, p < 0.001; GLM; Fig 3D). In Fig 3B–3D, size of *Arc*∩*H1a*(+) ensembles were also evaluated (MEC: A_240_-A_240_ vs. A_240_-A_10_: p < 0.001, F_(2,17)_ = 35.5, p < 0.001; LEC: A_240_-A_240_ vs. A_240_-A_10_: p < 0.001, F_(2,17)_ = 57.33, p < 0.001; CA3: A_240_-A_240_ vs. A_240_-A_10_: p = 0.185, F_(2,17)_ = 50.28, p < 0.001; 1-way ANOVA and Bonferroni correction). (E) Relationship between the size of *Arc*(+) ensembles and the length of exposure time for LEC (black), MEC (cyan), and CA3 (red) region on the cohort A_240_-, A_240_-A_10_, and A_240_-A_240_ was described and curve fitting was done. The standard error of fitted curve was depicted as shades. (F) Data of Fig 3E were normalized for the maximum size of *Arc*(+) ensemble in the 0–40 seconds. The estimated expansion of *Arc*(+) ensembles is depicted as exponential curve fitting (fitting parameters were described in Table 2). The standard error of fitted curve was depicted as shades.

We validated signal expression kinetics of RNAScope probes used in this study such as the Mm-Homer1a-tvS and the Mm-Arc-C3 described above. 3 animals stayed in homecage only were sacrificed and used as a negative control (S1 Fig). For detailed *H1a* and *Arc* signal kinetics, animals were exposed to the A_240_ and sacrificed after 4, 8, and 16 min (6 mice for each) from the end of A_240_ (S2 Fig). In this experiments, the tissue sections for MEC/LEC of CA3 were hybridized using both *Arc* and *H1a* riboprobes (Fig 3). Detailed methods for the *Arc/H1a* catFISH was described in manufacturer’s manual and previous studies [21]. The signal kinetics of intranuclear Arc and H1a were analyzed based on the results of S1 and S2 Figs.

### Image acquisition and analysis

**Fig 4:**
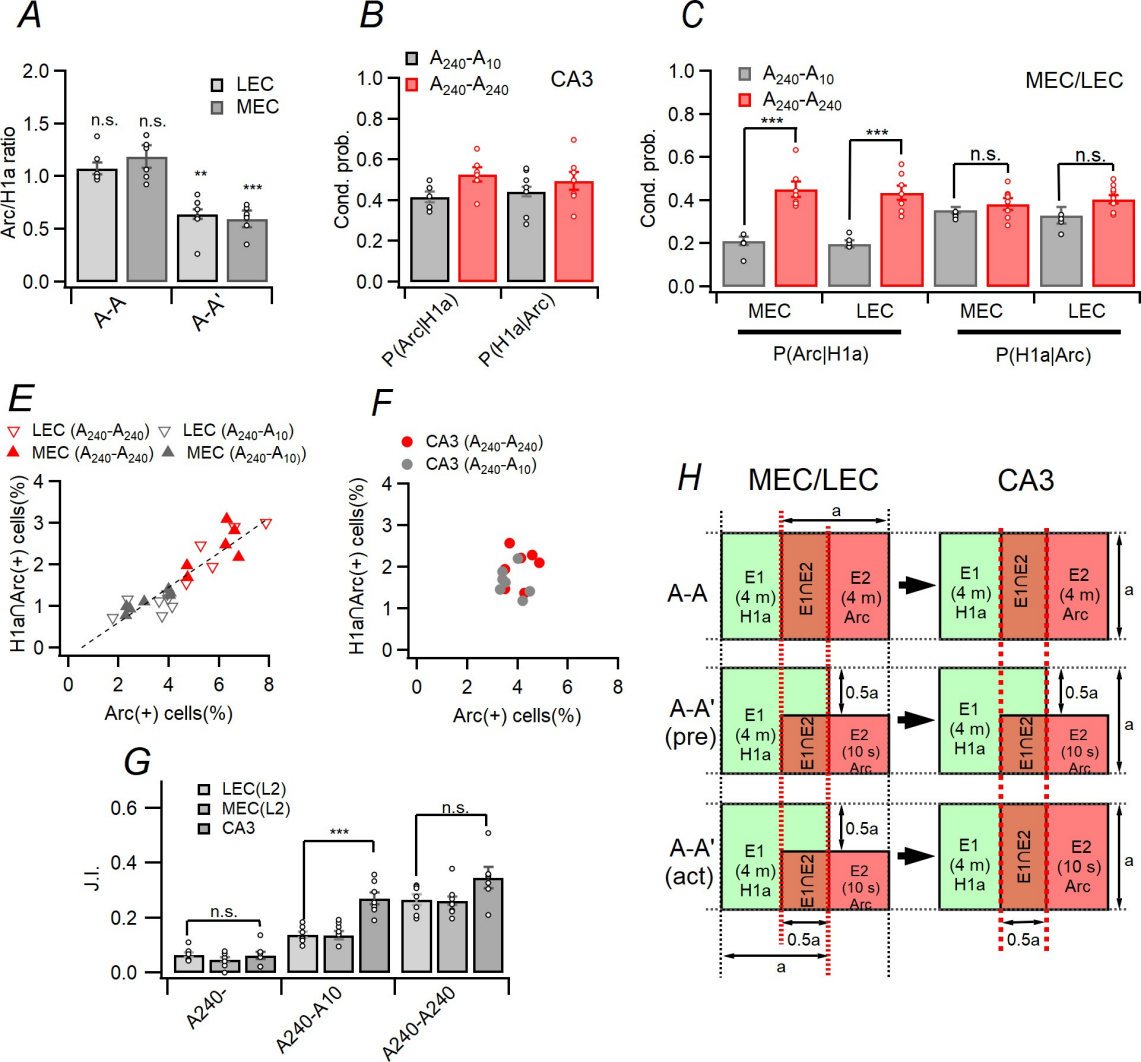
Analysis of ensembles for hippocampal CA3 and LEC/MEC region. (A) Ratio between *Arc*(+)/*H1a*(+) cells in MEC/LEC. This figure suggests that size of *Arc*(+) ensembles is about half of *H1a*(+) ensemble size for the cohort A_240_-A_10_. (B) Conditional probability, i.e. P(*Arc*|*H1a*) and P(*H1a*|*Arc*), in CA3. (C) The P(*Arc*|*H1a*) and P(*H1a*|*Arc*) in MEC/LEC (prob: F_(1,24)_ = 8.413, p = 0.008; cohort: F_(1,24)_ = 32.752, p < 0.001; region: F_(1,24)_ = 0.008, p = 0.929; cohort × prob × region: F_(1,24)_ = 3.576, p = 0.071; GLM). Because the size of the *Arc*(+) and *H1a*∩*Arc*(+) ensembles and was smaller than that of the *H1a*(+) ensembles, the values of P(*Arc*|*H1a*) are smaller in the cohort A_240_-A_10_ than in cohort A_240_-A_240_. (D) The linear relationship between the size of *Arc*(+) ensembles and *H1a*∩*Arc(*+) ensembles for LEC/MEC. Note that relationship between two variables are well fitted to linear function. (E) The relationship between the size of *Arc*(+) ensembles and *H1a*∩*Arc*(+) ensembles for the CA3. In the case of CA3, the size of the *Arc*(+) ensemble and *H1a*∩*Arc*(+) are distributed within a certain range regardless of the type of cohort. (F) *J*.*I*. between *Arc*(+) and *H1a*(+) ensembles in CA3 and MEC/LEC. Note that the *J*.*I*. on CA3 ensembles for A_240_-A_10_ cohort is much higher than that on MEC/LEC ensembles (region: F_(2,27)_ = 25.223, p < 0.001; cohort: F_(2,27)_ = 114.082, p < 0.001; cohort × region: F_(4,27)_ = 12.957, p < 0.001; GLM). (G) Schematic diagram of LEC/MEC and CA3 response evoked in A_240_-A_240_ (*upper*) and A_240_-A_10_ cohort (*middle* and *lower*). The middle row depicts that predicted CA3 responses (*right*) based on MEC/LEC activity (*left*) whereas the bottom low depicts that actual CA3 responses (*right*) based on MEC/LEC activity (*left*) which is evoked by novel exposure. Dotted red lines and their corresponding values indicate relative size of ensembles such as P(*H1a*∩*Arc*) for each cohorts and situations.

Hippocampal slices obtained from the animals were analyzed with *H1a/Arc* catFISH (Figs 2–4). Confocal z-stacks composed of 1-μm-thick optical sections were collected from the sagittal tissue sections containing MEC/LEC or CA3. Z-series of confocal images were obtained for the entire length of stratum pyramidale of CA3 area and the layer II of medial/lateral entorhinal cortex (hereafter MEC/LEC). The slides were photographed using TCS SP8 Dichroic/CS (Leica, Germany) and Olympus Fluoview FV1200 confocal microscope (Olympus, Japan) equipped with 488 and 633 nm diode lasers and their images were stored in a personal computer for further analysis. For signal detection, the settings for photomultiplier and laser power were optimized for detection of strong intranuclear signals and minimizing weaker cytoplasmic signals [23]. The number of intranuclear IEG signals was counted on 60× magnified photographs (N.A. = 1.2). Images were collected with a 60× objective to collect comparable numbers of cells from each sections. A single CA3-containing section and 1 LEC/MEC-containing section were analyzed for each animal. Non-neuronal cells, identified as small cells with intensely bright and uniformly stained nuclei, were excluded from the analysis. Only large, mottled nuclei present in the sections were regarded as neuronal cells, and included in the analysis. Neuronal nuclei were classified as negative (containing no transcription foci), *H1a*(+) (containing *H1a* foci), *Arc*(+) (containing *Arc* foci), or *Arc*∩*H1a*(+) (containing the foci for both *Arc* and *H1a*) by an experimenter blind to the classification of image stacks and the behavioral conditions they represented. Based on the expression kinetics of H1a/Arc INF described in the Figs 2 and 3, the *H1a*(+) neurons and *Arc*(+) neurons are those neurons that activated during the 0–4 min and 26–30 min of experimental session of Fig 2A, respectively. The *Arc*∩*H1a*(+) neurons are those activated at both 0–4 min and 26–30 min.

### Calculation of similarity between ensembles

As described previously [21], the size of *H1a*(+) or *Arc*(+) ensembles was calculated with the ratio of the number of *H1a*(+) or *Arc*(+) nuclei to the number of neuronal nuclei in certain region (i.e. a fraction of *H1a*(+) or *Arc*(+) nuclei) and denoted as P(*H1a*) or P(*Arc*) as necessary. The neural ensembles expressing both *Arc* and *H1a* is denoted as P(*H1a*∩*Arc*). In previous studies, measuring of pattern completion have been based on comparison of the similarity between ensembles [9, 15]. I calculated the similarity between E1 and E2 ensembles with Jaccard index (hereafter *J*.*I*.) [29]. The value of *J*.*I*. was calculated as follows:

$$J.I.=\frac{p(H1a\cap Arc)}{p(H1a\cup Arc)}$$

The conditional probability of *Arc* given *H1a*, P(*Arc*|*H*1*a*) is defined as P(*H*1*a*∩*Arc*)/P(*H*1*a*) and vice versa. Not only the J.I., but also the value of conditional probability was used to measure degree of overlap *H1a*(+) ensembles and *Arc*(+) ensembles (see Fig 4). The P(*Arc*|*H1a*) or P(*H1a*|*Arc*) are represented as the ratio between area of the rectangle corresponding to P(*H1a*∩*Arc*) and the rectangles corresponding to P*(H1a*) or P(*Arc*) which were denoted above, respectively. The *J*.*I*. are represented as the ratio between area of brown rectangle [i.e. P(*H*1*a*∩*Arc*)] and to total area of the shape enclosed by the solid outline [i.e P(*H*1*a*∪*Arc*)].

### Statistical analysis

The statistical data are expressed as mean ± standard error of the mean (SEM), and the number of cells/animals measured (details were described in results). Statistical data are evaluated for normality with Kolmogorov–Smirnov (K–S) test. For data that satisfy normality, statistical evaluations were performed with Student’s *t*-test, analysis of variance (ANOVA) or generalized linear model (GLM) and simple main effect analysis. Author did not use repeated measures ANOVA in this study because repeated measures of neural ensembles on the same animal cannot be performed with the *Arc/H1a* catFISH. For the brevity, the number of cells and statistical tests for determining statistical significance are stated in the text using following abbreviations: n.s.: no statistical significance; *: p < .05; **: p < .01; ***: p < .005. Statistical analyses were performed using PASW Statistics 18 (SPSS Inc, 2009, Chicago, IL).

## Results

### Pre-exposure mediated contextual fear conditioning (PECFC) of mice

Context pre-exposure facilitation effect (CPFE) is a phenomenon in which freezing response increase when a noxious stimulation is applied while re-exposed to the same context as the previously exposed before [24, 30]. The CPFC-based PECFC is impaired by the removal of CA3-PC synaptic output, suggesting the dependence of the PECFC on CA3 output [7]. Because the performance of PECFC depends upon the rapid acquisition of contextual learning and associational recall which is based on pattern completion [25], we used the PECFC and its modified protocol to evaluate pattern completion. Fig 1A summarized PECFC protocol in this study. Animals were exposed either the Ctx A or Ctx C on day 1. On day 2, all animals were exposed to a context-shock association pair. 11 animals were shocked after being exposed to the Ctx A for 10 seconds (6 animals: pre-exposed to Ctx A; 5 animals: pre-exposed to Ctx C), and 10 animals were shocked after being exposed to context A for 3 minutes (5 animls: pre-exposed to Ctx A; 5 animals: pre-exposed to Ctx C). All animals were returned to homecage after 30 seconds (Fig 1A). Animals that received shock after being exposed to the Ctx A for 3 minutes on day 2 showed freezing regardless of which context they were exposed to on day 1 (Ctx A vs. Ctx C: in 180+30 s: F_(1,17)_ = 0.171, p = 0.684, GLM and simple main effect analysis; Fig 1B). However, animals that received shock after being exposed to the Ctx A for 10 seconds on day 2 showed freezing only when they were pre-exposed to the Ctx A on day 1, and did not show freezing well when pre-exposed to the Ctx C (Ctx A vs. Ctx C in 10+30 s: F_(1,17)_ = 34.406, p < 0.001, GLM and simple main effect analysis; Fig 1B). In the case of the cohort exposed to the Ctx A on day 1, a similar level of freezing was shown regardless of the place-to-shock interval on day 2 (180+30 sec vs. 10+30 sec: F_(1,17)_ = 0.697, p = 0.415, GLM; Fig 1B). Although it has been proposed that PECFC have been predicted to depend on the hippocampal-dependent conjunctive recall [25] and Fig 1 suggests that degradation of retrieval cues and corresponding responses of animals would effectively evaluated by the PECFC, however, recall and pattern completion are completely different. Recall is a behavioral phenomenon and pattern separation is a neural process for which a cellular representation can be reinstated from partial or noisy cues [15, 31]. For compare similarities between cellular representations from whole or partial cues, measurement of CA3 or EC ensembles would be essential.

### Exposure of context pair for ensemble similarities

It is known that in order for an animal to associate an electric shock with a certain context, it must be exposed to the context for at least one minute before the shock is applied [30]. If an animal is exposed to electric shock immediately after exposure to a particular context, the animal does not exhibit freezing when subsequently exposed to the same context. However, if an animal had been exposed to a specific context before, it freezes even if it is exposed to a shock immediately after the exposure to the same context (CPFE) [24, 30]. Previous studies suggested that CPFE could be explained by conjunctive representation of patterns and retrieval of the entire pattern from a subset of stored patterns [25]. The process in the neuronal network by which a whole pattern is retrieved from a partial pattern is called as ‘pattern completion’. Results from previous studies and my current study revealed that the experience-dependent appearance of intranuclear foci (INF) of *Arc* (hereafter *Arc*-INF) is rapid and transient, and does not coincide with in time with the delayed experience-dependent appearance of the INF of *H1a* (Hereafter *H1a*-INF) [21, 23]. Experience of the A_240_ effectively induced the appearance of *Arc*-INF (red puncta in blue nuclei) in both LEC/MEC and CA3, but the *Arc*-INF disappeared in both CA3 and LEC/MEC areas 26 minutes after experience of the A_240_ (S1 Fig). Immediately after experience of the A_240_, *H1a*-INF (green puncta in blue nuclei) did not appear in both CA3 and LEC/MEC, but appeared in both CA3 and LEC/MEC 26 minutes after experience of the A_240_ (S1 Fig). There was no temporal overlap in the appearance of *H1a*-INF and *Arc*-INF in both the CA3 and LEC/MEC regions.

Based on the results of PECFC (Fig 1) and *Arc*/*H1a* FISH (S1 Fig) and previous studies [20], I constructed behavioral procedures for three cohorts (A_240_-, A_240_-A_10_, and A_240_-A_240_) to evaluate ensemble activities in the CA3 and LEC/MEC in response to the retrieval of spatial contexts as described in Fig 2A. The animal’s temporal position in the either A_240_ or A_10_ (Fig 2A) was recorded as video files and analyzed as heatmap. The cumulative travel length of the animals for A_240_ or A_10_ for the three cohorts is shown in Fig 2D. The cumulative travel length of animals is shorter in A_10_ than in A_240_, however, the gait speed does not significantly different between A_240_ and A_10_, regardless of the cohorts (cohort: F_(1,6)_ = 0.002, p = 0.970; context: F_(1,6)_ = 2.392, p = 0.173, cohort × context: F_(1,6)_ = 0.552, p = 0.486; repeated measures ANOVA; Fig 2E and Table 1). Combining the results of previous studies [7, 25, 32] and the Fig 1 of the current study, A_10_ could be considered as a degraded version of A_240_.

**Table 1:** Behavioral data of animals in three cohorts.

	travel length	gait speed	travel length	gait speed
	(m), 0–4 min	(cm/s), 0–4 min	(m), 26–30 min	(cm/s), 26–30 min
A_240_- (n = 6)	23.9 ± 1.63	9.9 ± 0.6	N/A	N/A
A_240_-A_10_ (n = 7)	21.9 ± 0.84	8.9 ± 0.5	1.0 ± 0.08	10.0 ± 1.3
A_240_-A_240_ (n = 7)	22.2 ± 0.56	9.2 ± 0.4	22.5 ± 0.9	9.6 ± 0.6

### Results of H1a/Arc catFISH on CA3 and MEC/LEC

Previous study and modelling have shown that PP input initiates recall and generalization of CA3 [6] and lesions of ECs cause loss of spatial memory retrieval [33]. Hippocampal CA3 region, which receives PP from LEC/MEC region, has an extensive recurrent network that would contribute to pattern completion [4, 6], a neural process that reinstate a whole representation from partial or noisy inputs [6]. Therefore, it would be important to measure both MEC (*left* column of Fig 3) / LEC (*middle* column of Fig 3) and CA3 (*right* column of Fig 3) ensembles in same animal and compare the neural ensembles which is evoked by the whole (for the cohort A_240_-A_240_; *top* row of Fig 3) and degraded inputs (for the cohort A_240_-A_10_; middle row of Fig 3). The cohort A_240_- was used as negative control for the *Arc* expression (bottom row of Fig 3).

Fig 3 shows the expression of *H1a* and *Arc* in the neurons as the mice explored during the behavioral session described in the Materials and Methods section and Fig 2. In the previous literature, it has been suggested and demonstrated that DG have little role in pattern completion, and the PP is important to initiate memory retrieval in CA3 [6, 33]. Since the cohort A_240_- remained on HC for 26 min after the A_240_ (see Fig 2), *Arc*-INF was barely expressed in both LEC/MEC and CA3 region as described in Fig 3 and S1 Table. The upper row of Fig 2 shows the representative figures of LEC (left), MEC (middle), and CA3 (right) ensembles for the mice of cohort A_240_-A_240_.

The size of *Arc*(+) ensemble is comparable with the size of *H1a*(+) ensemble in the mice of cohort A_240_-A_240_ in LEC/MEC (CA3: F_(1,34)_ = 1.249, p = 0.272; MEC: F_(1,34)_ = 3.66, p = 0.06; LEC: F_(1,34)_ = 0.587, p = 0.449; GLM and simple main effect analysis; Fig 3B–3D). In contrast of the cohort A_240_-A_240_, the size of *Arc*(+) ensembles of MEC/LEC is lower in the A_240_-A_10_ cohort than that the A_240_-A_240_ cohort (MEC: A_240_-A_240_ vs. A_240_-A_10_: p < 0.001; F_(2,34)_ = 48.02, p < 0.001; LEC: A_240_-A_240_ vs. A_240_-A_10_: p < 0.001; F_(2,34)_ = 39.71, p < 0.001; GLM and simple main effect analysis; Fig 3B and 3C). Based on the very short duration of environmental exposure in the A_10_, the size of *Arc*(+) ensemble in the LEC/MEC was smaller for the A_240_-A_10_ than the cohort A_240_-A_240_. This would be consistent with the results of previous studies and modeling describing the increased fear response of animal with the elongation of place-to-shock interval [18, 32] and the feature of EC ensembles that gradually adjusting their internal representations based on the change in external cues. In contrast of LEC/MEC, the size of *Arc*(+) ensemble in the CA3 in the A_240_-A_10_ cohort is similar to that of the A_240_-A_240_ cohort (CA3: A_240_-A_240_ vs. A_240_-A_10_: p < 0.001; F_(2,34)_ = 105.1, p < 0.001; GLM and simple main effect analysis). For the *Arc*∩*H1a*(+) ensembles, the size of *Arc*∩*H1a*(+) ensemble in the MEC/LEC of A_240_-A_10_ was smaller than that of the A_240_-A_240_, however, the size of *Arc*∩*H1a*(+) ensemble in the CA3 of A_240_-A_10_ was comparable with that of the A_240_-A_240_.

Before comparing the kinetics of the size of *Arc*(+) ensemble according to the exposed context in LEC/MEC and CA3, I compared The kinetics of *Arc*-INF and *H1a*-INF for various time delay conditions after exposure to the same context in LEC/MEC and CA3 (S2 Fig). The size of *Arc*(+) and *H1a*(+) ensemble (S2 Fig) were normalized based on the average of the maximum values. The detailed expression kinetics of *Arc*-INF and *H1a*-INF was not significantly different among the regions, such as for LEC, MEC and CA3.

For the CA3 and LEC/MEC, the size of neural ensembles for exposure time to the experimental contexts was verified through the size of *Arc*(+) ensembles by time (Fig 3E). Considering the results in the Fig 3 and S2 Fig, the slower *Arc*(+) expression kinetics of CA3 than that of LEC/MEC can be attributed to the gradual activation of *Arc*(+) ensembles in the LEC/MEC following gradual recognition of the components of external environment, rather than the delayed *Arc* mRNA expression in LEC/MEC. I examined the relationship between the expansion of the neural ensembles in CA3 or MEC/LEC and exposure time for the HC, A_10_, or A_240_ as described in Fig 3E and 3F. The size of the *Arc*(+) ensemble in CA3 had already approximated a maximum at 10 seconds of exposure, but that in MEC/LEC predicted to be approximated the maximum when exposed for at least 1 min. The expected expansion of the E2 ensembles according to the time was represented by exponential fitting (y = A+B*e*^-(*x*/*t*)^; see Table 2), As shown in Fig 3E and 3F. Considering that at least one minute of exploration is required to block the effect of CPFE [32], it can be assumed that exploring the same environment for 10 seconds would have been insufficient to perceive the entire environment and activate all neural ensembles corresponding to the entire input.

**Table 2:** Descriptive statistics of H1a/Arc ensembles in Fig 3.

		H1a	Arc	H1a ∩Arc	Curve fitting (3E)	Norm. Curve fitting (3F)
CA3	A_240_-	3.63 ± 0.21%	0.35 ± 0.04%	0.02 ± 0.04%	A = 3.96 ± 0.22	A = 1.00 ± 0.08
	A_240_-A_240_	3.94 ± 0.15%	3.75 ± 0.15%	1.63 ± 0.16%	B = -3.71 ± 0.32	B = -0.93 ± 0.08
	A_240_-A_10_	3.76 ± 0.09%	3.70 ± 0.30%	1.93 ± 0.12%	τ^-1^ = 0.26 ± 0.11	τ^-1^ = 0.26 ± 0.11
MEC (L II)	A_240_-	6.18 ± 0.44%	1.06 ± 0.26%	0.34 ± 0.10%	A = 6.79 ± 0.22	A = 1.00 ± 0.08
	A_240_-A_240_	5.36 ± 0.17%	3.17 ± 0.44%	1.10 ± 0.14%	B = -5.77 ± 0.70	B = -0.84 ± 0.10
	A_240_-A_10_	5.26 ± 0.48%	6.24 ± 0.70%	2.26 ± 0.23%	τ^-1^ = 0.06 ± 0.02	τ^-1^ = 0.06 ± 0.02
LEC (L II)	A_240_-	5.48 ± 0.20%	0.73 ± 0.10%	0.38 ± 0.05%	A = 6.21 ± 0.48	A = 1.00 ± 0.07
	A_240_-A_240_	5.50 ± 0.33%	3.34 ± 0.11%	1.04 ± 0.06%	B = -5.09 ± 0.68	B = -0.82 ± 0.11
	A_240_-A_10_	6.33 ± 0.44%	6.85 ± 0.66%	2.80 ± 0.33%	τ^-1^ = 0.08 ± 0.02	τ^-1^ = 0.08 ± 0.02

### Verification of pattern completion in CA3 and EC circuits using catFISH

I compared the size of the *H1a*(+) and *Arc*(+) ensembles as an Arc/H1a ratio in MEC/LEC, as shown in Fig 4A. Thus, the size of *H1a* ensembles are similar as or slightly larger than that of *Arc* ensembles in the cohort A_240_-A_240_ (MEC: t = 1.313, p = 0.237; LEC: t = 2.619, p = 0.04; one-sample t-test; Fig 4A), however, in cohort A_240_-_A10,_ the size of *Arc*(+) ensembles are significantly smaller than *H1a*(+) ensembles (MEC: t = –5.506, p = 0.012; LEC: t = –6.098, p = 0.009; one-sample t-test). I calculated P(*Arc*|*H1a*) and P(*H1a*|*Arc*) in CA3 and MEC/LEC (Fig 4B and 4C; based on the results in the Fig 3). The P(*Arc*|*H1a*) and P(*H1a*|*Arc*) of CA3 in A_240_-A_240_ is slightly larger than that in A_240_-A_10_ and (cohort: F_(1,24)_ = 5.09, p = 0.03, probe: F_(1,24)_ = 0.004, p = 0.949; cohort × probe: F_(1,24)_ = 0.683, p = 0.417; GLM; Fig 4B). The value of P(*Arc*|*H1a*) in the cohort A_240_-A_10_ (about 0.2) was significantly smaller than that in the cohort A_240_-A_240_ (about 0.4), on both of the LEC/MEC (MEC: F_(1,48)_ = 39.387, p < 0.001; LEC: F_(1,48)_ = 39.333, p < 0.001; GLM and simple main effect analysis; Fig 4C). However, the value of P(*H1a*|*Arc*) was not significantly different between the cohorts on both of the MEC/LEC (MEC: F_(1,48)_ = 0.539, p = 0.466; LEC: F_(1,48)_ = 3.818, p = 0.057; GLM and simple main effect analysis; Fig 4C). I compared the size of *Arc*(+) ensembles and the *H1a*∩*Arc*(+) ensembles in MEC/LEC. The overall relationship between the size of *Arc*(+) ensembles and that of *H1a*∩*Arc*(+) ensembles was well fitted into linear relationship in both LEC and MEC, regardless of the cohorts. Also, size of two ensembles was proportional to the exposure time of the external environment (slope: 0.42 ± 0.03, p < 0.001; y-intercept: -0.22 ± 0.178, p = 0.227; R^2^ = 0.86; Fig 4D). However, in the CA3 region, the size of *Arc*(+) ensembles and *H1a*∩*Arc*(+) did not significantly changed regardless of exposure time of the external environment (Fig 4E). For estimate overlap between *H1a*(+) and *Arc*(+) ensembles based on the conditional probability and similarity index, I calculated the similarity between the *H1a*(+) and *Arc*(+) ensembles with Jaccard index (hereafter J.I.) [39] for the three cohorts as showed in the Fig 4G. For the cohort A_240_-A_240_ (F_(2,18)_ = 2.31, p = 0.128, 1-way ANOVA) and A_240_- (F_(2,18)_ = 0.721, p = 0.502, 1-way ANOVA), the value of J.I. in CA3 is not significantly different between the MEC/LEC and CA3. However, for the cohort A_240_-A-_10_, the value of J.I. of CA3 was significantly higher than that of MEC/LEC (MEC vs. CA3: p < 0.001, LEC vs. CA3: p < 0.001, MEC vs. LEC: p = 1.00; F_(2,18)_ = 17.87, p < 0.001, 1-way ANOVA and Bonferroni correction; Fig 4F). In order to compare ensemble similarities between regions and arrange them as shown in Fig 4G below, only comparisons within the same cohort are required, so 1-way ANOVA would be sufficient for comparison in Fig 4F.

In Fig 4G, I predicted the sizes of *H1a*(+), *Arc*(+) and *H1a∩Arc*(+) ensembles in the LEC/MEC and CA3 regions for the A_240_-A_10_ cohort by dividing them into cases with (*lower* row of Fig 4H) and without (*middle* row of Fig 4H) an auto-associational network in CA3. The *H1a*(+) or Arc(+) ensemble of each region is represented by a square with ’a’ in green and red, respectively, and therefore the sizes of *H1a*(+) or *Arc*(+) ensembles in were corresponded to a^2^. Note that a^2^ is not an absolute value, but represents the relative size of the ensemble for each region based on the cohort A_240_-A_240_. The J.I. was represented with a ratio between the area of brown rectangle [i.e. P(*H1a*∩*Arc*)] and the area of figures enclosed by black solid line [i.e. P(*H1a*∪*Arc*)]. For example, the J.I. of MEC/LEC ensembles and CA3 ensembles in the cohort A_240_-A_10_ was calculated as about 0.2 (= 0.25a^2^/1.25a^2^) and 0.33 (= 0.5a^2^/1.5a^2^), respectively, as described in the Fig 4G and the middle row of 4H. For the cohort A_240_-A_240_, the J.I of MEC/LEC ensembles and CA3 ensembles was calculated as about 0.33, as described in the Fig 4G and the upper row of 4H. Based on the results of the Fig 4D-4F, Venn diagrams of the size of *Arc*(+) ensemble in EC can be drawn as described in the Fig 4G.

Considering the results of this study, it can be inferred that a short exposure (10 seconds) to a certain environment only activated a subset of the entire ensembles that would have been activated by a longer exposure (4 minutes) and could be drawn as a small rectangle in Fig 4G (*middle* row). Previous studies showed that PP projects onto the distal dendrites of CA3-PC would be diffuse and distributed in SLM of CA3 region with laminar fashion [18]. Based on the previous studies, I could assume that if CA3 were had not auto-association network, the size of the *Arc*(+) ensembles in CA3 evoked by the cohort A_240_-A_10_ (which represents partial inputs) would be much smaller than that evoked by the cohort A_240_-A_240_ (which represents whole inputs). However, in LEC/MEC, the *Arc*(+) ensemble size of cohort A_240_-A_10_ was smaller than that of A_240_-A_240_, but in CA3, the *Arc*(+) ensemble size was similar in both cohort A_240_-A_10_ and cohort A_240_-A_240_. These results are consistent with the previous study [41], which showed that focal stimulation of stratum radiatum in CA3 propagated to activate whole networks in CA3. So, I could assume that activation of subset of CA3 ensembles and subsequent activation of a whole CA3 ensembles activated by the partial inputs from EC.

## Discussion

### Difference between previous studies and present study

Although pattern completion in CA3 auto-associational network have been widely studied in previous studies and reviews [9, 10, 13, 14, 34], these studies have limitations in that they did not distinguish between pattern convergence and pattern completion [14] and did not deal with the precise definition of pattern completion [15]. Moreover, previous studies did not compare the size of ensembles and similarities between ensembles in EC evoked by whole cues and degraded cues [9, 35]. For comparison of ensembles induced by whole cues or reduced cues, I had applied a behavioral experiment based on CPFE, a phenomenon in which the freezing response increases as the time delay between noxious stimuli after exposure increases (Figs 1 and 2) [24, 25, 32]. Another previous studies showed that when the output of CA3-PC was ablated but the direct cortical input to CA3 was normal, rapid one-trial learning was suppressed but incremental learning by repetitive trials was not suppressed [7]. This would be consistent with previous suggestions for opposing features of the hippocampus (rapid formation of discrete representations) and neocortex (incremental adjustment of representations in response to external inputs) [36, 37]. Results in this study would support that the size of EC ensembles induced by degraded cues would be existing between the size of EC ensembles induced by whole cues and the EC ensemble when not exposed to any cues. Considering that the similarity of the two ensembles corresponding to each context would be important for recognizing two contexts [38], the difference of PEFC results shown in previous papers could be attributed to differences in CA3 internal expression induced by pre-exposure and re-exposure [7, 22].

### Evaluation of pattern completion in CA3 and EC with molecular method

I showed that kinetics of *Arc* and *H1a* riboprobes used in this study were the same as those of *Arc* and *H1a* riboprobes used in previous studies [20, 23]. Also, the expression kinetics of *Arc* riboprobe were similar in the EC and CA3 regions. This would mean that the smaller size of the EC ensemble than that of the CA3 ensemble after exposure to A_10_ would not because the Arc mRNA expression rate differs by region, but rather that it would be due to the gradual adjustment of the entorhinal cortex by external inputs. Unlike the hippocampal proper, the changes in the internal expression of the EC are known to gradually change in response to changes in the external environment [13]. Because the PP projection from EC layer II onto distal dendrites of CA3-PCs has laminar structure [18], more sparse activation of EC circuits *per se* evoked by A_10_ would activate only a subset of the CA3 ensembles evoked by A_240_. However, the size of CA3 ensembles evoked by the A_10_ was not different significantly as that evoked by the A_240_ (Figs 3 and 4). A previous study revealed that circuit response pattern in CA3 region which was induced by dual-site LTP induction protocol was readily reproduced through the stimulation of only single site [39]. Considering the expression of hippocampal *H1a* was triggered by the induction of LTP [40] and inhibition of hippocampal *Arc* disrupts maintenance of LTP and memory consolidation [41], it was conceivable that an internal representation would be formed in LEC/MEC and CA3 during the exposure of 0–4 min, and it would be reactivated in the exposure of 26–30 min (Fig 2A). Although exposure for 10 seconds in the exposure of 26–30 min (Fig 2A) would activated only a part of the EC internal representation and a part of the CA3 internal representation (Fig 3A), it can be assumed that the activated CA3 subset would activate the rest of the representations as well, considering the previous studies and models (Fig 4) [3, 39, 42]. Although the previous paper used pattern separation as an antonym of pattern completion [13, 14], it should be noted that the antonym of pattern separation is clearly different as pattern convergence.

### Limitations of this study

This study has several limitations. Although the CPFE (Fig 1) and derived protocols (Figs 2 and 3) would be useful for evaluating rapid one-trial contextual learning, blockade of cholinergic projection with muscarinic receptor antagonist showed impairment of contextual learning performance [24]. Although evaluation of encoding could be evaluated by the size of H1a(+) ensembles, considering that cholinergic activity would be important for the hippocampal encoding and consolidation [43], it would be worth that the possibility that not only retrieval but also encoding would be involved in performance of CPFE. Also, even if the experiment was performed using a sufficient number of animals, there may be limitations in sufficient interpretation of neural functions and animal cognitive or behavior. This means that it was necessary to pay attention to the interpretation of the results of this experiment. Although DG ensembles could dramatically change by slight difference between the contexts [13], activity of granule cell could potentiate CA3-PC excitability and facilitates PP-LTP induction [21, 44]. Thus, the potential influence of MF activity to CA3 ensembles could not be ruled out, although its potential influence would be small.

## Conclusion

The present study directly compared EC ensembles would be evoked by partial cue inputs and whole cue inputs and demonstrated the detailed differences. Also I showed amplification of CA3-projected EC outputs would facilitate the activation of the whole representation of CA3 ensembles.

## Supporting information

S1 Fig Expression of Arc/H1a signals in CA3 and EC layer II. (A) Schedule of the procedure for t = 0 m (*upper*) and t = 26 m (*lower*). For the t = 0 m cohort, mice were exposed the A_240_ and sacrificed immediately. For the t = 26 m cohort, mice were exposed to A_240_ and kept in homecage (HC) for 26 min until sacrificed. (B) In mice that were only in HC, few *H1a* and *Arc* signals were detected in CA3, MEC, and LEC as described in the S1 Table. (C) After the mice of t = 0 m group (*left* column) and t = 26 m (*right* column) group were sacrificed, *H1a*/*Arc* catFISH was performed and the results were observed in CA3 (*upper* column), MEC (*middle* column), and LEC (*right* column). Regardless of the region, *Arc*-INF expression was rapid and transient, prominent only at t = 0 m and almost absent at HC and t = 26 m. On the other hand, *H1a*-INF expression was almost absent at HC and t = 0 m and high only at t = 26 m. (D) The experience-dependent appearance of Arc-INF and H1a-INF in the CA3 was summarized (*Arc*: HC vs. t = 26 min: p = 0.721, HC vs. t = 0 min: p < 0.001, t = 0 min vs. t = 26 min: p < 0.001; *H1a*: HC vs. t = 26 min: p = 0.457, HC vs. t = 0 min: p < 0.001, t = 0 min vs. t = 26 min: p < 0.001; GLM and simple main effect analysis). (E) Experience-dependent appearance of *Arc*-INF and *H1a*-INF in the MEC was summarized (*Arc*: HC vs. t = 26 min: p = 0.128, HC vs. t = 0 min: p < 0.001, t = 0 min vs. t = 26 min: p < 0.001; *H1a*: HC vs. t = 26 min: p = 0.749, HC vs. t = 0 min: p < 0.001, t = 0 min vs. t = 26 min: p < 0.001; GLM and simple main effect analysis). (F) The experience-dependent appearance of *Arc*-INF and *H1a*-INF in the LEC was summarized (*Arc*: HC vs. t = 26 min: p = 0.527, HC vs. t = 0 min: p < 0.001, t = 0 min vs. t = 26 min: p < 0.001; *H1a*: HC vs. t = 26 min: p = 0.412, HC vs. t = 0 min: p < 0.001, t = 0 min vs. t = 26 min: p < 0.001; GLM and simple main effect analysis). The size of H1a(+) and Arc(+) ensembles for each time was summarized in Table 1 (riboprobes: F_(1,72)_ = 0.237, p = 0.628, region: F_(2,72)_ = 15.292, p < 0.001, time: F_(2,72)_ = 0.004, p < 0.001, riboprobes × region: F_(2,72)_ = 0,629, p = 0.536, riboprobes × region × time: F_(4,72)_ = 8.164, p < 0.001; GLM and simple main effect analysis).
(TIF)

S2 Fig Kinetics of intranuclear Arc and H1a signals was measured. (A) Experiment schedule for 4 m (*upper*), 4 m + 4 m (*middle*) and 4 m + 12 m (*lowe*_r_). For 4 m cohort, mice were exposed the A_240_ and sacrificed immediately (adopted from the t = 0 m cohort of S1 Fig). For the 4 m + 4 m cohort, mice were exposed A_240_ and left in HC for 4 min until sacrifice. For the 4 m + 12 m cohort, mice were exposed A_240_ and left in HC for 12 min until the sacrifice. (B) After the mice of the 4 m cohort (*upper* row), 4 m + 4 m cohort (*middle* row) and 4 m + 12 m cohort (*lower* row) were sacrificed, H1a/Arc catFISH was performed. Results are shown in S2 Fig as follows: MEC (*left* column), LEC (*middle* column) and CA3 (*right* column). Regardless of the region, *Arc*-INF expression was prominent at the 4 m (immediately after A_240_) and diminished at the 4 m + 4 m (4 min after A_240_) and almost absent at the 4 m + 12 m (12 min after A_240_). *H1a*-INF expression was almost absent at the 4 m, 4 m + 4 m and 4 m + 12 m cohorts regards of regions. The H1a-NF expression was delayed until the 26 min after the exposure of A_240_ (refer S1 Fig). (C) Experience-dependent appearance of *Arc*-INF was peaked immediately after A_240_, and declined as time elapsed. At 26 minutes after the A_240_, the *Arc*-INF expression decreased a level comparable to that of HC. The appearance of *H1a*-INF was not significant until 26 minutes after A_240_, in contrast of the *Arc*-INF. (D) Kinetics of experience-dependent appearance of Arc-INF and H1a-INF was normalized with the mean peak value of *Arc*-INF (4 m) and *H1a*-INF ensemble size (4 min + 26 min; adopted from S1 Fig). The expression kinetics of *Arc*-INF (region: F_(2,66)_ = 2.160, p = 0.123, time: F_(4,66)_ = 273.4, p < 0.001; GLM) and *H1a*-INF (region: F_(2,66)_ = 0.677, p = 0.512, time: F_(4,66)_ = 518.0 p < 0.001; GLM) as lapse of time was not significantly different among the CA3, MEC and LEC. The appearance of Arc-INF was not coinciding in time with the delayed appearance of H1a-INF.
(TIF)

S1 Table Kinetics of H1a/Arc signals in CA3 and MEC/LEC. (DOCX)

S2 Table Detailed kinetics of normalized Arc/H1a ensemble size in EC layer II and CA3 as lapse of time. (DOCX)

S1 Data (XLSX)

## References

[1] Rebola N, Carta M, Mulle C. Operation and plasticity of hippocampal CA3 circuits: implications for memory encoding. Nat Rev Neurosci. 2017;18(4):208–20. doi: 10.1038/nrn.2017.10 28251990

[2] Hunsaker MR, Kesner RP. The operation of pattern separation and pattern completion processes associated with different attributes or domains of memory. Neurosci Biobehav Rev. 2013;37(1):36–58. doi: 10.1016/j.neubiorev.2012.09.014 23043857

[3] Marr D. Simple memory: a theory for archicortex. Philos Trans R Soc Lond B Biol Sci. 1971; 262,:23–81. doi: 10.1098/rstb.1971.0078 4399412

[4] Amaral D, Ishizuka N, Claiborne B. Neurons, numbers and the hippocampal network. Progress in Brain Research. 1990;83:1–11. doi: 10.1016/s0079-6123(08)61237-6 2203093

[5] McNaughton BL, Morris RG. Hippocampal synaptic enhancement and information storage within a distributed memory system. Trends in neurosciences. 1987;10(10):8.

[6] Rolls ET. The mechanisms for pattern completion and pattern separation in the hippocampus. Front Syst Neurosci. 2013;7:74. doi: 10.3389/fnsys.2013.00074 24198767 PMC3812781

[7] Nakashiba T, Young JZ, McHugh TJ, Buhl DL, Tonegawa S. Transgenic inhibition of synaptic transmission reveals role of CA3 output in hippocampal learning. Science. 2008;319(5867):1260–4. doi: 10.1126/science.1151120 18218862

[8] Nakazawa K, Sun L, Quirk M, Rondi-Reig L, Wilson M, Tonegawa S. Hippocampal CA3 NMDA Receptors Are Crucial for Memory Acquisition of One-Time Experience. Neuron. 2003;38:305–15. doi: 10.1016/s0896-6273(03)00165-x 12718863

[9] Neunuebel J, Knierim J. CA3 retrieves coherent representations from degraded input: direct evidence for CA3 pattern completion and dentate gyrus pattern separation. Neuron. 2014;81(2):416–27. doi: 10.1016/j.neuron.2013.11.017 24462102 PMC3904133

[10] Grande X, Berron D, Horner AJ, Bisby JA, Duzel E, Burgess N. Holistic Recollection via Pattern Completion Involves Hippocampal Subfield CA3. J Neurosci. 2019;39(41):8100–11. doi: 10.1523/JNEUROSCI.0722-19.2019 31405925 PMC6786823

[11] Cholvin T, Hainmueller T, Bartos M. The hippocampus converts dynamic entorhinal inputs into stable spatial maps. Neuron. 2021;109(19):3135–48 e7. doi: 10.1016/j.neuron.2021.09.019 34619088 PMC8516433

[12] Moser MB, Rowland DC, Moser EI. Place cells, grid cells, and memory. Cold Spring Harb Perspect Biol. 2015;7(2):a021808. doi: 10.1101/cshperspect.a021808 25646382 PMC4315928

[13] Leutgeb JK, Leutgeb S, Moser MB, Moser EI. Pattern separation in the dentate gyrus and CA3 of the hippocampus. Science. 2007;315(5814):961–6. doi: 10.1126/science.1135801 17303747

[14] Yassa MA, Stark CEL. Pattern separation in the hippocampus. Trends in Neurosciences. 2011;34(10):515–25. doi: 10.1016/j.tins.2011.06.006 21788086 PMC3183227

[15] Santoro A. Reassessing pattern separation in the dentate gyrus. Front Behav Neurosci. 2013;7:96. doi: 10.3389/fnbeh.2013.00096 23908611 PMC3726960

[16] Miao C, Cao Q, Ito HT, Yamahachi H, Witter MP, Moser MB, et al. Hippocampal Remapping after Partial Inactivation of the Medial Entorhinal Cortex. Neuron. 2015;88(3):590–603. doi: 10.1016/j.neuron.2015.09.051 26539894

[17] Latuske P, Kornienko O, Kohler L, Allen K. Hippocampal Remapping and Its Entorhinal Origin. Front Behav Neurosci. 2017;11:253. doi: 10.3389/fnbeh.2017.00253 29354038 PMC5758554

[18] Witter MP, Doan TP, Jacobsen B, Nilssen ES, Ohara S. Architecture of the Entorhinal Cortex A Review of Entorhinal Anatomy in Rodents with Some Comparative Notes. Front Syst Neurosci. 2017;11:46. doi: 10.3389/fnsys.2017.00046 28701931 PMC5488372

[19] Witter MP. Organization of the entorhinal-hippocampal system: A review of current anatomical data. Hippocampus. 1993;3:12. 8287110

[20] Vazdarjanova A, Guzowski JF. Differences in hippocampal neuronal population responses to modifications of an environmental context: evidence for distinct, yet complementary, functions of CA3 and CA1 ensembles. J Neurosci. 2004;24(29):6489–96. doi: 10.1523/JNEUROSCI.0350-04.2004 15269259 PMC6729865

[21] Eom K, Lee HR, Hyun JH, An H, Lee YS, Ho WK, et al. Gradual decorrelation of CA3 ensembles associated with contextual discrimination learning is impaired by Kv1.2 insufficiency. Hippocampus. 2022;32(3):193–216. doi: 10.1002/hipo.23400 34964210

[22] Nakazawa K, Quirk MC, Chitwood RA, Watanabe M, Yeckel MF, Sun LD, et al. Requirement for hippocampal CA3 NMDA receptors in associative memory recall. Science. 2002;297(5579):211–8. doi: 10.1126/science.1071795 12040087 PMC2877140

[23] Vazdarjanova A, McNaughton BL, Barnes CA, Worley PF, Guzowski JF. Experience-dependent coincident expression of the effector immediate-early genes arc and Homer 1a in hippocampal and neocortical neuronal networks. J Neurosci. 2002;22:5. doi: 10.1523/JNEUROSCI.22-23-10067.2002 12451105 PMC6758761

[24] Brown KL, Kennard JA, Sherer DJ, Comalli DM, Woodruff-Pak DS. The context preexposure facilitation effect in mice: a dose-response analysis of pretraining scopolamine administration. Behav Brain Res. 2011;225(1):290–6. doi: 10.1016/j.bbr.2011.07.044 21827794 PMC3179919

[25] Rudy JW O’Reilly RC. Conjunctive representations, the hippocampus, and contextual fear conditioning. Cognitive, Affective, & Behavioral Neuroscience. 2001;1:17. doi: 10.3758/cabn.1.1.66 12467104

[26] Pennington ZT, Dong Z, Feng Y, Vetere LM, Page-Harley L, Shuman T, et al. ezTrack: An open-source video analysis pipeline for the investigation of animal behavior. Sci Rep. 2019;9(1):19979. doi: 10.1038/s41598-019-56408-9 31882950 PMC6934800

[27] Bottai D, Guzowski JF, Schwarz MK, Kang SH, Xiao B, Lanahan A, et al. Synaptic Activity-Induced Conversion of Intronic to Exonic Sequence in Homer 1 Immediate Early Gene Expression. The Journal of Neuroscience. 2002;22(1):167–75. doi: 10.1523/JNEUROSCI.22-01-00167.2002 11756499 PMC6757601

[28] Eom K, Lee HR. Measuring Pattern Separation in Hippocampus by in Situ Hybridization. Curr Protoc. 2022;2(8):e522. doi: 10.1002/cpz1.522 35980141

[29] Jaccard P. The Distribution of the Flora in the Alpine Zone.1. New Phytologist. 1912;11:14.

[30] Fanselow MS. Factors governing one-trial contextual conditioning. Animal Learning & Behavior. 1990;18:264.

[31] Hersman S, Rodriguez Barrera V, Fanselow M. Assigning Function to Adult-Born Neurons: A Theoretical Framework for Characterizing Neural Manipulation of Learning. Front Syst Neurosci. 2015;9:182. doi: 10.3389/fnsys.2015.00182 26778981 PMC4700131

[32] Fanselow M. Associative vs topographical accounts of the immediate shock-freezing deficit in rats: Implications for the response selection rules governing species-specific defensive reactions. Learning and Motivation. 1986;17(1):14.

[33] Lee I, Kesner RP. Encoding versus retrieval of spatial memory: double dissociation between the dentate gyrus and the perforant path inputs into CA3 in the dorsal hippocampus. Hippocampus. 2004;14(66–76). doi: 10.1002/hipo.10167 15058484

[34] McHugh TJ, Jones MW, Quinn JJ, Balthasar N, Coppari R, Elmquist JK, et al. Dentate gyrus NMDA receptors mediate rapid pattern separation in the hippocampal network. Science. 2007;317(5834):94–9. doi: 10.1126/science.1140263 17556551

[35] Knierim JJ, Neunuebel JP. Tracking the flow of hippocampal computation: Pattern separation, pattern completion, and attractor dynamics. Neurobiol Learn Mem. 2016;129:38–49. doi: 10.1016/j.nlm.2015.10.008 26514299 PMC4792674

[36] McClelland J, Goddard N. Considerations arising from a complementary learning systems perspective on hippocampus and neocortex. Hippocampus. 1996;6:12. doi: 10.1002/(SICI)1098-1063(1996)6:6<654::AID-HIPO8>3.0.CO;2-G 9034852

[37] Norman KA. How hippocampus and cortex contribute to recognition memory: revisiting the complementary learning systems model. Hippocampus. 2010;20(11):1217–27. doi: 10.1002/hipo.20855 20857486 PMC3416886

[38] Czerniawski J, Guzowski JF. Acute neuroinflammation impairs context discrimination memory and disrupts pattern separation processes in hippocampus. J Neurosci. 2014;34(37):12470–80. doi: 10.1523/JNEUROSCI.0542-14.2014 25209285 PMC4160778

[39] Jackson MB. Recall of spatial patterns stored in a hippocampal slice by long-term potentiation. J Neurophysiol. 2013;110(11):2511–9. doi: 10.1152/jn.00533.2013 24027100 PMC3882773

[40] Hoang TH, Boge J, Manahan-Vaughan D. Hippocampal subfield-specific Homer1a expression is triggered by learning-facilitated long-term potentiation and long-term depression at medial perforant path synapses. Hippocampus. 2021;31(8):897–915. doi: 10.1002/hipo.23333 33964041

[41] Guzowski JF, McNaughton BL, Barnes CA, Worley PF. Environment-specific expression of the immediate-early gene Arc in hippocampal neuronal ensembles. Nat Neurosci. 1999;2:5. 10570490 10.1038/16046

[42] Rolls ET. The storage and recall of memories in the hippocampo-cortical system. Cell Tissue Res. 2018;373(3):577–604. doi: 10.1007/s00441-017-2744-3 29218403 PMC6132650

[43] Hasselmo ME. The role of acetylcholine in learning and memory. Curr Opin Neurobiol. 2006;16(6):710–5. doi: 10.1016/j.conb.2006.09.002 17011181 PMC2659740

[44] Hyun J, Eom K, Lee K, Bae J, Bae Y, Kim M, et al. Kv1.2 mediates heterosynaptic modulation of direct cortical synaptic inputs in CA3 pyramidal cells. J Physiol. 2015;593(16):3617–43. doi: 10.1113/JP270372 26047212 PMC4560587

