# Supplementary figures and images for: Partial EC outputs by degraded cues are amplified in hippocampal CA3 circuits for retrieving stored patterns
Source: PLoS One. 2023 Apr 19;18(4):e0281458. doi: 10.1371/journal.pone.0281458 (PMC10115257; doi:10.1371/journal.pone.0281458)

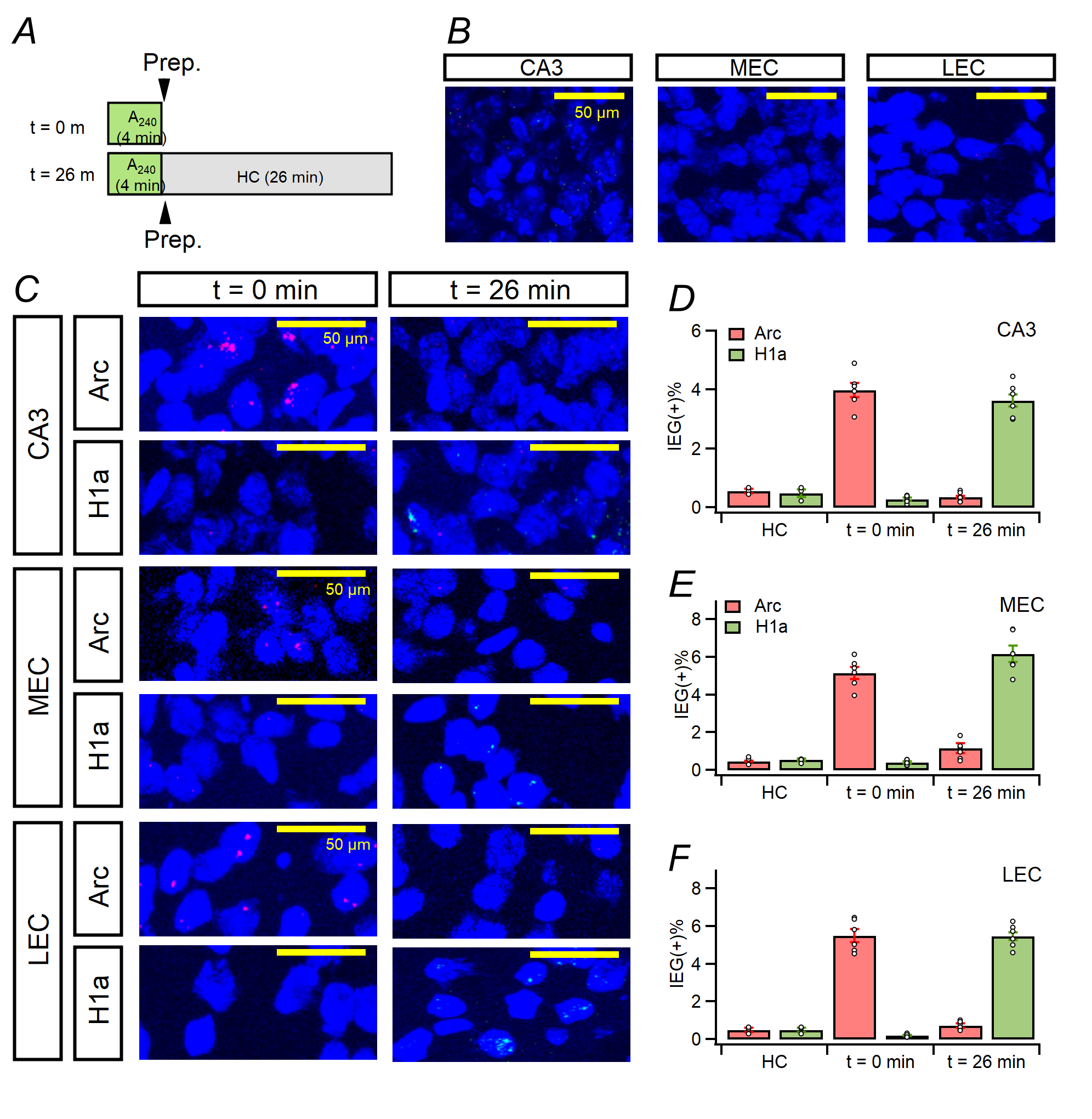

Supplement: S1 Fig — (A) Schedule of the procedure for t = 0 m (upper) and t = 26 m (lower). For the t = 0 m cohort, mice were exposed the A240 and sacrificed immediately. For the t = 26 m cohort, mice were exposed to A240 and kept in homecage (HC) for 26 min until sacrificed. (B) In mice that were only in HC, few H1a and Arc signals were detected in CA3, MEC, and LEC as described in the S1 Table. (C) After the mice of t = 0 m group (left column) and t = 26 m (right column) group were sacrificed, H1a/Arc catFISH was performed and the results were observed in CA3 (upper column), MEC (middle column), and LEC (right column). Regardless of the region, Arc-INF expression was rapid and transient, prominent only at t = 0 m and almost absent at HC and t = 26 m. On the other hand, H1a-INF expression was almost absent at HC and t = 0 m and high only at t = 26 m. (D) The experience-dependent appearance of Arc-INF and H1a-INF in the CA3 was summarized (Arc: HC vs. t = 26 min: p = 0.721, HC vs. t = 0 min: p < 0.001, t = 0 min vs. t = 26 min: p < 0.001; H1a: HC vs. t = 26 min: p = 0.457, HC vs. t = 0 min: p < 0.001, t = 0 min vs. t = 26 min: p < 0.001; GLM and simple main effect analysis). (E) Experience-dependent appearance of Arc-INF and H1a-INF in the MEC was summarized (Arc: HC vs. t = 26 min: p = 0.128, HC vs. t = 0 min: p < 0.001, t = 0 min vs. t = 26 min: p < 0.001; H1a: HC vs. t = 26 min: p = 0.749, HC vs. t = 0 min: p < 0.001, t = 0 min vs. t = 26 min: p < 0.001; GLM and simple main effect analysis). (F) The experience-dependent appearance of Arc-INF and H1a-INF in the LEC was summarized (Arc: HC vs. t = 26 min: p = 0.527, HC vs. t = 0 min: p < 0.001, t = 0 min vs. t = 26 min: p < 0.001; H1a: HC vs. t = 26 min: p = 0.412, HC vs. t = 0 min: p < 0.001, t = 0 min vs. t = 26 min: p < 0.001; GLM and simple main effect analysis). The size of H1a(+) and Arc(+) ensembles for each time was summarized in Table 1 (riboprobes: F(1,72) = 0.237, p = 0.628, region: F(2,72) = 15.292, p < 0.001, time: F(2,72 [file pone.0281458.s001.tif]

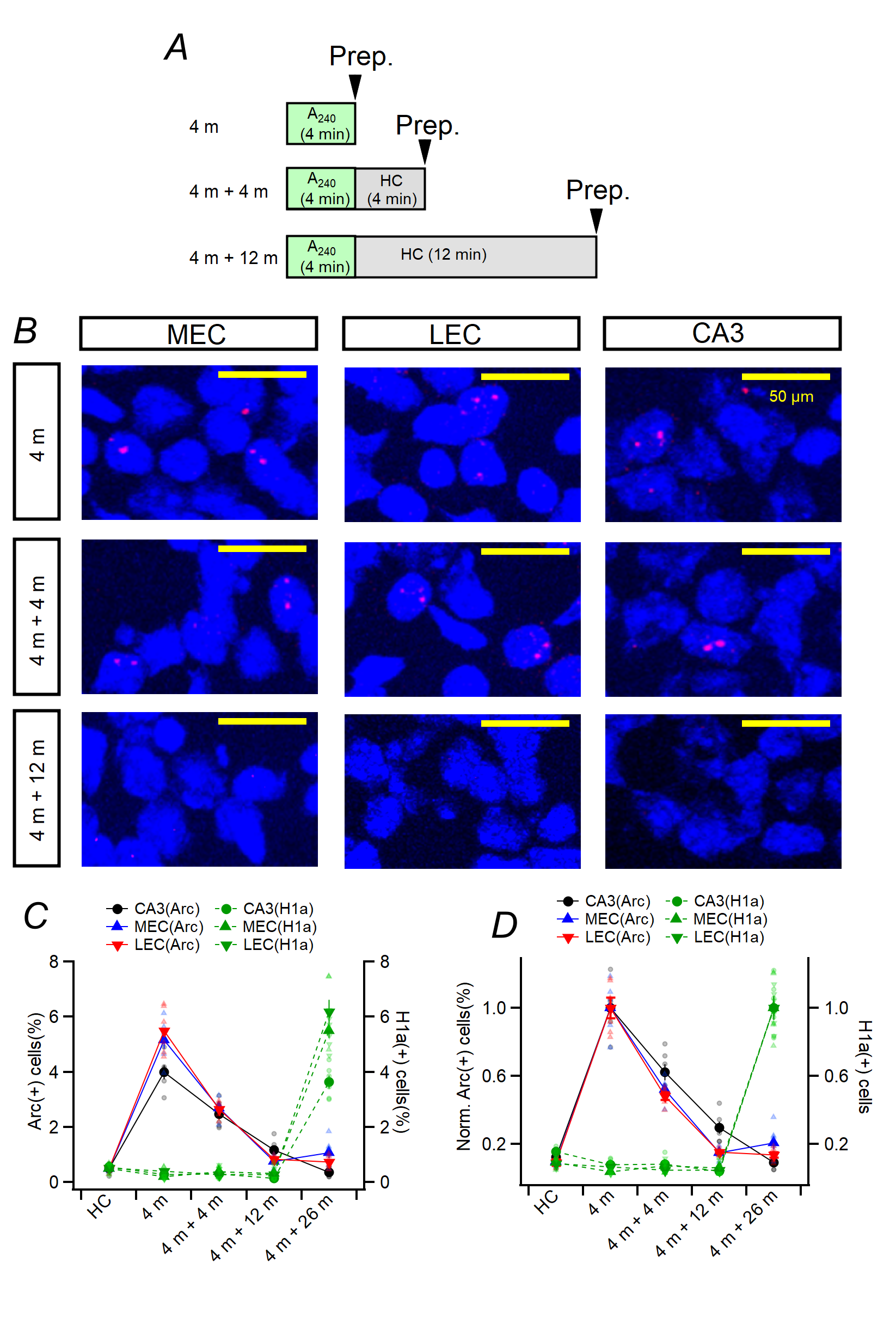

Supplement: S2 Fig — (A) Experiment schedule for 4 m (upper), 4 m + 4 m (middle) and 4 m + 12 m (lower). For 4 m cohort, mice were exposed the A240 and sacrificed immediately (adopted from the t = 0 m cohort of S1 Fig). For the 4 m + 4 m cohort, mice were exposed A240 and left in HC for 4 min until sacrifice. For the 4 m + 12 m cohort, mice were exposed A240 and left in HC for 12 min until the sacrifice. (B) After the mice of the 4 m cohort (upper row), 4 m + 4 m cohort (middle row) and 4 m + 12 m cohort (lower row) were sacrificed, H1a/Arc catFISH was performed. Results are shown in S2 Fig as follows: MEC (left column), LEC (middle column) and CA3 (right column). Regardless of the region, Arc-INF expression was prominent at the 4 m (immediately after A240) and diminished at the 4 m + 4 m (4 min after A240) and almost absent at the 4 m + 12 m (12 min after A240). H1a-INF expression was almost absent at the 4 m, 4 m + 4 m and 4 m + 12 m cohorts regards of regions. The H1a-NF expression was delayed until the 26 min after the exposure of A240 (refer S1 Fig). (C) Experience-dependent appearance of Arc-INF was peaked immediately after A240, and declined as time elapsed. At 26 minutes after the A240, the Arc-INF expression decreased a level comparable to that of HC. The appearance of H1a-INF was not significant until 26 minutes after A240, in contrast of the Arc-INF. (D) Kinetics of experience-dependent appearance of Arc-INF and H1a-INF was normalized with the mean peak value of Arc-INF (4 m) and H1a-INF ensemble size (4 min + 26 min; adopted from S1 Fig). The expression kinetics of Arc-INF (region: F(2,66) = 2.160, p = 0.123, time: F(4,66) = 273.4, p < 0.001; GLM) and H1a-INF (region: F(2,66) = 0.677, p = 0.512, time: F(4,66) = 518.0 p < 0.001; GLM) as lapse of time was not significantly different among the CA3, MEC and LEC. The appearance of Arc-INF was not coinciding in time with the delayed appearance of H1a-INF. (TIF) [file pone.0281458.s002.tif]
