# Supplementary material for: Partial EC outputs by degraded cues are amplified in hippocampal CA3 circuits for retrieving stored patterns
Source: PLoS One. 2023 Apr 19;18(4):e0281458. doi: 10.1371/journal.pone.0281458 (PMC10115257; doi:10.1371/journal.pone.0281458)
Supplement: S1 Table — (DOCX) [file pone.0281458.s003.docx]

|  | | *H1a* | *Arc* |
| --- | --- | --- | --- |
| CA3 | HC (n = 3) | 0.56 ± 0.06% | 0.48 ± 0.14% |
|  | t = 0 min (n = 7) | 0.28 ± 0.04% | 3.98 ± 0.25% |
|  | t = 26 min (n = 7) | 3.63 ± 0.20% | 0.35 ± 0.04% |
| MEC  (Layer II) | HC (n = 3) | 0.51 ± 0.12% | 0.49 ± 0.07% |
|  | t = 0 min (n = 7) | 0.39 ± 0.06% | 5.15 ± 0.31% |
|  | t = 26 min (n = 7) | 6.18 ± 0.44% | 1.06 ± 0.25% |
| LEC  (Layer II) | HC (n = 3) | 0.59 ± 0.36% | 0.58 ± 0.36% |
|  | t = 0 min (n = 7) | 0.19 ± 0.03% | 5.49 ± 0.34% |
|  | t = 26 min (n = 7) | 5.48 ± 0.20% | 0.73 ± 0.10% |
