# Supplementary material for: Partial EC outputs by degraded cues are amplified in hippocampal CA3 circuits for retrieving stored patterns
Source: PLoS One. 2023 Apr 19;18(4):e0281458. doi: 10.1371/journal.pone.0281458 (PMC10115257; doi:10.1371/journal.pone.0281458)
Supplement: S2 Table — (DOCX) [file pone.0281458.s004.docx]

|  | | *Arc* | *H1a* |
| --- | --- | --- | --- |
| CA3 | HC | 0.12 ± 0.03 | 0.15 ± 0.02 |
|  | 4 m | 1.00 ± 0.06 | 0.07 ± 0.13 |
|  | 4 m + 4 m | 0.62 ± 0.05 | 0.08 ± 0.02 |
|  | 4 m + 12 m | 0.29 ± 0.04 | 0.04 ± 0.01 |
|  | 4 m + 26 m | 0.09 ± 0.02 | 1.00 ± 0.06 |
| MEC (LII) | HC | 0.10 ± 0.01 | 0.09 ± 0.02 |
|  | 4 m | 1.00 ± 0.06 | 0.04 ± 0.01 |
|  | 4 m + 4 m | 0.52 ± 0.03 | 0.07 ± 0.01 |
|  | 4 m + 12 m | 0.15 ± 0.01 | 0.06 ± 0.01 |
|  | 4 m + 26 m | 0.21 ± 0.04 | 1.00 ± 0.06 |
| LEC (LII) | HC | 0.09 ± 0.02 | 0.08 ± 0.02 |
|  | 4 m | 1.00 ± 0.06 | 0.06 ± 0.01 |
|  | 4 m + 4 m | 0.48 ± 0.02 | 0.04 ± 0.01 |
|  | 4 m + 12 m | 0.15 ± 0.01 | 0.05 ± 0.01 |
|  | 4 m + 26 m | 0.13 ± 0.02 | 1.00 ± 0.07 |
